# Baseline Whole-Gland MRI Radiomics for Risk Stratification After Suspicious mpMRI and Negative Biopsy: A MULTIPROS Sub-Analysis

**DOI:** 10.3390/cancers18172823

**Published:** 2026-09-01

**Authors:** Wafa D. Aloufi, Magdalena Szewczyk-Bieda, Asma Aloufi, Cheng Wei, Luigi Manfredi

**Affiliations:** 1 Division of Imaging Sciences and Technology, School of Medicine, University of Dundee, Ninewells Hospital, Dundee DD1 9SY, UK; 2389778@dundee.ac.uk (W.D.A.); c.wei@dundee.ac.uk (C.W.); 2 Department of Radiological Sciences, College of Applied Medical Sciences, Taif University, Taif 21944, Saudi Arabia; 3 Department of Clinical Radiology, Ninewells Hospital and Medical School, Dundee DD1 9SY, UK; magdalena.szewczyk-bieda@nhs.scot; 4 Department of Computer Science, College of Computers and Information Technology, Taif University, Taif 21944, Saudi Arabia; aeoufi@tu.edu.sa; 5 Division of Respiratory Medicine and Gastroenterology, School of Medicine, University of Dundee, Ninewells Hospital, Dundee DD1 9SY, UK

**Keywords:** radiomics, prostate cancer, multiparametric MRI, clinically significant prostate cancer, negative biopsy, TRUS, follow-up, prediction model, machine learning

## Abstract

**Background/Objectives**: Patients with suspicious prostate mpMRI but negative baseline biopsy remain challenging to manage, as some are later diagnosed with clinically significant prostate cancer (csPCa). This study evaluated whether baseline whole-gland radiomic features from T2-weighted imaging and apparent diffusion coefficient (ADC) maps could predict subsequent csPCa detection. **Methods**: This retrospective radiomics sub-analysis of the MULTIPROS trial included men with suspicious baseline mpMRI findings (PI-RADS v2.0 categories 3–5) and no csPCa detected at baseline biopsy. The primary outcome was subsequent csPCa detection during follow-up, defined as ISUP Grade Group ≥2. Whole-gland prostate segmentation was performed on baseline T2-weighted images using a semi-automated approach with manual refinement, and the resulting T2-derived masks were applied to the spatially corresponding ADC maps. Radiomic features were extracted using PyRadiomics version 3.1.0a2 after standardised preprocessing. Feature robustness was assessed in a 20-patient reproducibility subset using intra- and inter-reader intraclass correlation coefficients, followed by correlation filtering and LASSO-based feature prioritisation. Radiomics-only, clinicoradiological-only, and clinicoradiological–radiomic logistic regression models were evaluated using stratified five-fold cross-validation. **Results**: T2-based modelling included 151 patients, of whom 36/151 (23.8%) had subsequent csPCa detected; the median time to csPCa detection or last follow-up was 64 months. ADC analyses included 144 patients, of whom 34/144 (23.6%) had subsequent csPCa detected. The T2 clinicoradiological–radiomic model achieved a mean AUC of 0.696 ± 0.060, compared with 0.713 ± 0.072 for the T2 clinicoradiological-only model and 0.456 ± 0.091 for the T2 radiomics-only model. In the ADC-available cohort, ADC radiomics-only, ADC clinicoradiological–radiomic, and combined T2 + ADC clinicoradiological–radiomic models achieved mean AUCs of 0.684 ± 0.052, 0.635 ± 0.157, and 0.663 ± 0.106, respectively. Calibration was imperfect, with deviations at higher predicted probabilities and overprediction in the highest predicted-risk bin. **Conclusions**: Baseline whole-gland MRI radiomics showed limited and inconsistent predictive value for subsequent csPCa detection after negative biopsy and did not demonstrate consistent incremental improvement over clinicoradiological predictors. These findings are exploratory and hypothesis-generating, and external validation is required before clinical use.

## Simple Summary

Men with suspicious prostate MRI findings but a negative biopsy can be difficult to manage because some are later diagnosed with clinically significant prostate cancer. This study tested whether quantitative MRI features, known as radiomic features, could help estimate the likelihood of subsequent clinically significant prostate cancer detection during available follow-up. We analysed whole-gland prostate MRI features from patients in the MULTIPROS study who had suspicious MRI findings but negative baseline biopsy. Overall, radiomics added little to standard clinical and imaging predictors such as PSA density, MRI score, lesion location, and atypical biopsy findings. These results suggest that whole-gland MRI radiomics may capture some prostate imaging variation, but its added value was limited. The model should not be used clinically without testing in larger independent patient groups.

## 1. Introduction

Prostate cancer is the second most commonly diagnosed cancer and the fifth leading cause of cancer death among men worldwide [1]. Multiparametric MRI (mpMRI) is now central to the diagnostic pathway for suspected prostate cancer, supporting lesion detection, biopsy targeting, local staging, and risk stratification using PI-RADS [2,3,4]. However, a clinically important subgroup consists of patients with suspicious mpMRI findings but negative targeted and/or systematic biopsy results. In this setting, uncertainty remains because a negative biopsy does not fully exclude csPCa, and the optimal follow-up strategy remains unclear [5,6]. This represents a recognised diagnostic and management dilemma.

Current follow-up decisions in this subgroup are commonly guided by clinical judgement, PSA-based risk assessment, PSA density, repeat mpMRI, and selective repeat biopsy [2,7]. Although clinically useful, these approaches may not fully capture the baseline imaging phenotype of the prostate. Radiomics has been proposed as a method for extracting quantitative imaging features related to intensity, shape, and texture, which may capture imaging heterogeneity; however, the biological and clinical meaning of these features requires further validation [8]. Recent systematic evidence suggests that prostate MRI radiomics shows promise for csPCa prediction, although clinical applicability remains limited by methodological heterogeneity, inconsistent superiority over PI-RADS assessment, and insufficient prospective and external validation [9].

Although prostate MRI radiomics has been widely studied for baseline csPCa detection, lesion characterisation, and tumour grading [10,11,12,13,14], its role in risk stratification after suspicious mpMRI and negative baseline biopsy remains poorly defined. This post-negative-biopsy setting is clinically important because patients may remain at risk of subsequently detected csPCa despite an initially negative histological result.

This MULTIPROS sub-analysis addressed this gap by evaluating whether baseline whole-gland radiomic features derived from T2-weighted images and ADC maps could predict subsequent csPCa detection during long-term follow-up. We hypothesised that radiomic features, particularly when combined with clinicoradiological predictors, might provide complementary risk-stratification information in this clinically challenging population.

## 2. Materials and Methods

### 2.1. Study Design and Population

This retrospective radiomics study was conducted as a sub-analysis of the MULTIPROS prospective randomised trial, whose study design and primary diagnostic outcomes have been previously published [15,16]. The present analysis included men with suspicious baseline mpMRI findings, defined as PI-RADS v2.0 categories 3–5 [17], with no csPCa detected at baseline biopsy and available clinical follow-up. Among patients without subsequent csPCa detection, at least 12 months of available follow-up was required; patients with csPCa detected within 12 months were retained. The eligible follow-up cohort comprised 153 patients. After excluding two patients with low-quality T2-weighted images, 151 were included in T2-based modelling, while ADC-based and combined T2 + ADC analyses were performed in the 144 patients with available ADC maps. No separate a priori sample size calculation was performed for this retrospective radiomics sub-analysis. The sample size was determined by the available eligible MULTIPROS participants who met the study inclusion criteria and had images of sufficient quality for radiomic analysis. This sub-analysis had Caldicott approval IGTCAL-2024-167; the MULTIPROS trial was registered at ClinicalTrials.gov (NCT02745496) [15,16].

### 2.2. Baseline Biopsy, Follow-Up, and Outcome

Baseline biopsy was performed according to the MULTIPROS protocol, as previously described [15,16]. Briefly, patients underwent systematic biopsy alone or combined MRI/US fusion-targeted and systematic biopsy according to trial allocation.

After a negative baseline biopsy, patients underwent clinical follow-up with PSA monitoring and clinical review. Follow-up was not protocolised uniformly across participants. Repeat MRI and/or biopsy were not protocol-mandated and were performed according to clinical concern, including radiological–pathological discordance, ASAP, rising PSA, interval lesion progression, or multidisciplinary team decision. When repeat biopsy was performed for subsequent diagnostic assessment, biopsy approaches included systematic and, where clinically indicated, MRI-targeted sampling; repeat-biopsy procedures were not standardised as part of the study protocol. Repeat biopsy could be undertaken with or without an intervening repeat MRI. No subsequent csPCa diagnosis in the present radiomics cohort was made through prostatectomy or another surgical pathway.

Patients were followed until subsequent csPCa detection or their last recorded clinical review, with follow-up data collected through May 2025. Time to subsequent csPCa detection or last follow-up was calculated from the date of the baseline mpMRI. The primary outcome was subsequent histological detection of csPCa, defined as ISUP Grade Group ≥2. Because follow-up investigations were clinically driven, absence of detected csPCa did not necessarily represent definitive exclusion of disease.

### 2.3. MRI Acquisition and Preparation

Baseline mpMRI was acquired before biopsy according to the MULTIPROS imaging protocol [15,16]. MRI examinations were performed across the participating centres using a 3-T Magnetom Trio-PrismaFIT system (Siemens Healthineers, Erlangen, Germany; NHS Tayside), a 3-T Achieva system (Philips Healthcare, Best, The Netherlands; NHS Grampian), and a 1.5-T Ingenia system (Philips Healthcare, Best, The Netherlands; Royal Free London). Baseline mpMRI examinations were prospectively interpreted as part of the original MULTIPROS trial using PI-RADS v2.0. For the present radiomics analysis, axial T2-weighted images and ADC maps were used. All radiomic analyses were performed using baseline pre-biopsy MRI only. Repeat MRI examinations acquired during follow-up were not used for segmentation, radiomic feature extraction, feature selection, or model development. Images were anonymised using a Python workflow with pydicom and converted from DICOM/IMA to compressed NIfTI format using dcm2niix. Spatial correspondence between T2-weighted images and ADC maps was assessed visually and, where required, by checking image geometry, bounding-box overlap, and mutual information after ADC resampling to the T2 reference geometry. Geometry or mask discrepancies were addressed before radiomic feature extraction using corrected masks or PyRadiomics mask correction where appropriate. During PyRadiomics feature extraction, images were resampled to isotropic 1 × 1 × 1 mm voxels using B-spline interpolation, while corresponding binary masks were resampled using nearest-neighbour interpolation. Segmentation was performed on the baseline T2-weighted images only. For ADC-based radiomic analysis, the T2-derived whole-gland masks were applied to the spatially corresponding ADC maps after placement of the ADC images on the T2 reference grid; no separate ADC segmentation was performed. T2-weighted images underwent N4 bias-field correction followed by robust z-score intensity normalisation using non-background voxels. Normalisation statistics were calculated after excluding extreme voxel intensities outside the 1st–99th percentile range, and the resulting z-score images were clipped to −5 to +5. PyRadiomics image-level normalisation was disabled during feature extraction. T2-weighted images were discretised using a fixed bin width of 0.1, while ADC maps were retained on their quantitative intensity scale and discretised using a fixed bin width of 25. ComBat harmonisation was not applied. The smallest acquisition group comprised only five cases acquired at 1.5 T, limiting the reliability of statistical harmonisation across acquisition groups.

### 2.4. Segmentation

Whole-gland prostate segmentation was performed in ITK-SNAP using a semi-automated approach, followed by manual review and refinement. Whole-gland segmentation was used because the study aimed to assess patient-level risk stratification after a negative baseline biopsy rather than to classify an individual histologically confirmed lesion. Masks were saved as binary NIfTI volumes, with the prostate gland labelled as 1 and background as 0.

Segmentation repeatability and reproducibility were assessed in a randomly selected subset of 20 patients with both T2-weighted imaging and ADC maps available; no formal sample-size calculation was performed for this subset. The subset size was selected pragmatically for an exploratory reproducibility assessment. The researcher (W.D.A.) repeated the segmentation after a two-week interval, and the radiologist (M.S.B.) independently segmented the same cases. Spatial agreement was evaluated using the Dice similarity coefficient and 95th percentile Hausdorff distance. Feature-level reproducibility was assessed using ICC(A,1), a two-way absolute-agreement, single-measurement ICC. Features were retained only if they achieved an ICC ≥ 0.75 in both the intra-reader and inter-reader comparisons; features failing either criterion were excluded before subsequent correlation filtering and model development. Pairwise Spearman correlation filtering was performed using the same 20-patient reproducibility subset, with redundancy defined as |ρ| ≥ 0.90.

### 2.5. Feature Extraction and Reduction

Radiomic features were extracted separately from T2-weighted images and ADC maps using PyRadiomics with predefined sequence-specific YAML settings provided as Supplementary File S1. PyRadiomics normalisation was disabled because T2-weighted images had already undergone external N4 correction and robust z-score normalisation, whereas ADC maps were retained on their quantitative intensity scale. Only the PyRadiomics Original image type was enabled for radiomic feature extraction; no filtered or transformed image types were used. Extracted feature families included first-order, shape, GLCM, GLRLM, GLSZM, GLDM, and NGTDM features. Features satisfying the predefined ICC and correlation criteria in the 20-patient reproducibility subset were retained for modelling.

LASSO logistic regression was used to prioritise candidate radiomic features separately for T2-weighted and ADC imaging. An outer stratified five-fold cross-validation procedure was used. Within each outer training fold, predictors were standardised using parameters estimated from that training fold. The inverse regularisation parameter, C, was selected by grid search over values of 0.001, 0.005, 0.01, 0.05, 0.1, 0.5, 1, 5, and 10 using inner stratified five-fold cross-validation, with ROC-AUC as the optimisation criterion. LASSO was fitted using an L1 penalty, the LIBLINEAR solver, balanced class weights, a maximum of 5000 iterations, and a random seed of 42. The corresponding outer test fold was not used during scaling, penalty selection, or LASSO fitting.

Features with non-zero LASSO coefficients were recorded within each outer training fold, and their selection frequencies across the five folds were calculated. LASSO was used to prioritise candidate features rather than to determine the final feature set automatically. Given the limited number of csPCa events, the final radiomic predictors were defined using an explicit rule-based prioritisation step: candidate features were ranked by selection frequency across the five outer folds, and features with the highest selection frequency were then prioritised according to larger absolute standardised LASSO coefficients, complementary feature classes, and model parsimony. One T2 feature and two ADC features were retained for final model development. Because selection frequencies across all outer folds informed the final fixed feature sets, the subsequent cross-validated performance assessment was not fully independent of feature definition and was considered exploratory.

### 2.6. Model Development

Logistic regression was selected as the primary modelling approach because of its interpretability, suitability for the modest sample size, and compatibility with the limited number of outcome events. Clinicoradiological-only, radiomics-only, and clinicoradiological–radiomic models were developed. Radiomics-only models included only the final retained radiomic predictors for the relevant MRI sequence and did not include clinicoradiological variables. Clinicoradiological–radiomic models combined these retained radiomic predictors with the predefined clinicoradiological predictor set. The clinicoradiological predictor set comprised baseline PSA density, PI-RADS 4, PI-RADS 5, transition-zone lesion location, anterior fibromuscular stroma lesion location, and ASAP status. PI-RADS 3, peripheral-zone location, and absence of ASAP were used as reference categories. Baseline PSA and prostate volume were not included together with PSA density to avoid redundancy.

The final T2 radiomic predictor was original_gldm_DependenceNonUniformityNormalized. The final ADC radiomic predictors were original_glcm_Imc1 and original_firstorder_RootMeanSquared. These predictors were held fixed during final model evaluation.

Logistic-regression models used L2 regularisation with the LBFGS solver, a maximum of 5000 iterations, and a random seed of 42. The inverse regularisation parameter, C, was tuned within each outer training fold using inner stratified five-fold cross-validation over values of 0.001, 0.005, 0.01, 0.05, 0.1, 0.5, 1, 5, and 10, with ROC-AUC as the optimisation criterion. The primary analysis used unweighted logistic regression. Class-weighted logistic regression was evaluated as a sensitivity analysis. Continuous predictors were imputed and standardised within each training fold, while binary predictors were retained as 0/1 variables.

Excluding the intercept, the T2 clinicoradiological-only, T2 radiomics-only, and T2 clinicoradiological–radiomic models contained 6, 1, and 7 predictors, respectively. The ADC clinicoradiological-only, ADC radiomics-only, ADC clinicoradiological–radiomic, and combined T2 + ADC clinicoradiological–radiomic models contained 6, 2, 8, and 9 predictors, respectively.

Linear SVM and shallow decision-tree classifiers were evaluated as simple supplementary comparator models using the same final T2 clinicoradiological–radiomic predictor set, representing alternative linear-margin and rule-based approaches while avoiding unnecessary model complexity in this small-event setting. Hyperparameter tuning was performed within the inner cross-validation loop of each outer training fold. The linear SVM used a linear kernel with probability estimation, with C tuned over 0.001, 0.005, 0.01, 0.05, 0.1, 0.5, 1, 5, and 10. The decision tree was tuned over maximum depth values of 1, 2, and 3; minimum samples per leaf of 5, 10, and 15; and minimum samples for split of 10 and 20. These restricted hyperparameter ranges were intentionally chosen to balance limited model flexibility with overfitting control in a relatively small dataset. The primary classifier comparison used unweighted models, with class-weighted logistic regression evaluated separately as a sensitivity analysis.

### 2.7. Internal Validation and Performance Assessment

Final model performance was evaluated using stratified five-fold cross-validation. The selected radiomic features were held fixed across evaluation folds. For logistic regression and linear SVM, the inverse regularisation parameter, C, was selected by inner stratified five-fold cross-validation within the corresponding outer training fold. For the decision tree, maximum depth, minimum samples per leaf, and minimum samples for split were tuned within the same inner cross-validation framework. Missing continuous values were median-imputed within each training fold, while missing binary values, if any, were imputed using the most frequent category. Continuous predictors were standardised for logistic regression and linear SVM, but not for the decision tree, using training-fold parameters only. The held-out outer fold was used solely for performance evaluation.

Threshold-dependent metrics were calculated using thresholds derived from the outer training data. Specifically, inner out-of-fold predicted probabilities within the outer training fold were used to identify the Youden threshold, which was then applied to the corresponding held-out outer test fold. A fixed 0.50 threshold was evaluated as a supplementary sensitivity analysis. These thresholds were exploratory and were not validated clinical thresholds for repeat biopsy.

Model performance was assessed using AUC, accuracy, balanced accuracy, sensitivity, specificity, precision, F1 score, Youden index, and confusion-matrix counts, and was summarised as mean ± standard deviation across folds. PPV and NPV were additionally calculated for the fixed 0.50 and training-derived Youden thresholds; means were calculated only across folds in which the corresponding predictive value was defined. Because the final fixed feature sets were informed by selection frequencies calculated across all outer folds during the preceding feature-prioritisation stage, the final performance estimates were considered exploratory and may contain some optimism. Pooled out-of-fold AUCs were reported with 95% confidence intervals estimated using 20,000 stratified patient-level bootstrap resamples of the pooled out-of-fold predictions. For key incremental-value comparisons within the same cohort, paired bootstrap 95% confidence intervals were calculated for differences in pooled out-of-fold AUC using identical patient resamples for both models. Cross-cohort statistical comparisons were not performed. A training-only feature-selection sensitivity analysis was also performed to assess potential optimism from the fixed radiomic feature definition. Within each outer training fold, LASSO feature selection was repeated using training data only, retaining one T2 and two ADC features as in the primary models. The corresponding outer test fold remained uninvolved in feature selection and model fitting.

Calibration of the primary T2 clinicoradiological–radiomic model was evaluated using pooled out-of-fold predicted probabilities, a five-bin quantile-based calibration curve, Brier score, calibration intercept, and calibration slope. Reporting was guided by TRIPOD + AI [18] and CLEAR [19]. Radiomic feature extraction used PyRadiomics with IBSI-consistent feature definitions [20].

### 2.8. Statistical Analysis

Baseline characteristics were summarised for the overall cohort and according to subsequent csPCa detection status. Continuous variables were assessed as non-normally distributed and are therefore reported as the median with interquartile range and compared using the Mann–Whitney U test. Categorical variables are reported as counts and percentages and compared using the chi-square test or Fisher’s exact test, as appropriate.

Radiomic feature extraction was performed in an Anaconda environment using Python 3.9.23, PyRadiomics version 3.1.0a2, and SimpleITK version 2.5.2. Statistical analysis, feature prioritisation, model development, internal validation, calibration assessment, and figure generation were performed separately using Python version 3.13. ICC assessment and correlation filtering were performed using the 20-patient reproducibility subset. During nested LASSO feature prioritisation, standardisation, LASSO fitting, and regularisation-parameter tuning were restricted to the outer training folds. During final model evaluation, the selected radiomic features were held fixed, while standardisation, model fitting, and threshold determination were performed using the training data within each cross-validation fold. The held-out data were used only to estimate model performance. A random seed of 42 was used for reproducibility.

## 3. Results

### 3.1. Cohort Characteristics

**Figure 1:**
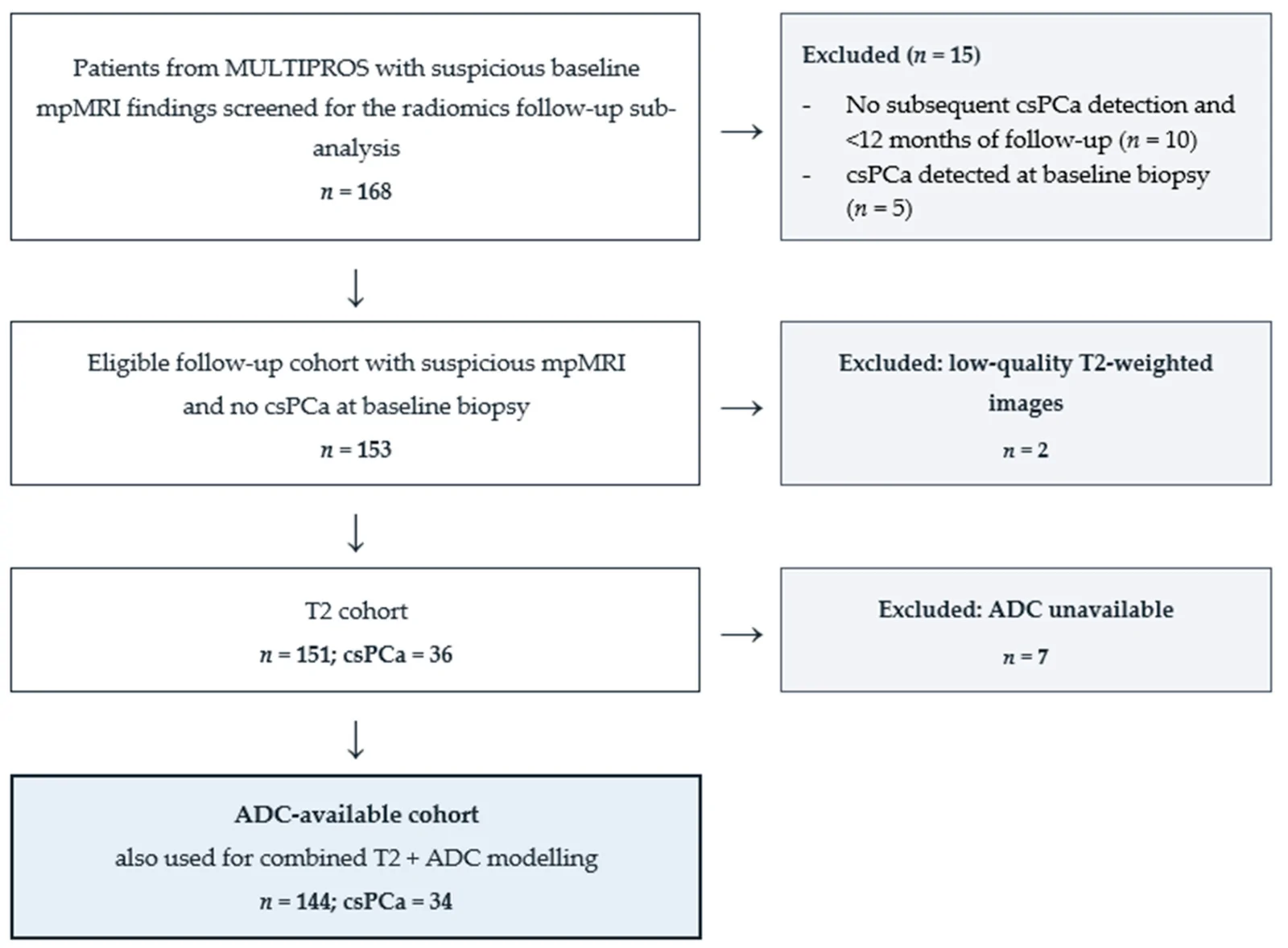
Study design and participant flow diagram for the radiomics sub-analysis.

The derivation of the final T2 and ADC-available cohorts is shown in Figure 1. In the T2 modelling cohort, 151 patients were included, of whom 36 (23.8%) had subsequent csPCa detected during follow-up, while 115 (76.2%) had no csPCa detected during the available follow-up period. Baseline characteristics are summarised in Table 1. Patients with subsequent csPCa detection had significantly smaller prostate volumes, higher PSA density, different PI-RADS score distribution, different lesion-location distribution, and a higher frequency of ASAP compared with those without csPCa.

Age and baseline PSA level did not differ significantly between groups. The median time to subsequent csPCa detection was 3 months (IQR 2–6).

### 3.2. Segmentation Reproducibility and Feature Selection

**Figure 2:**
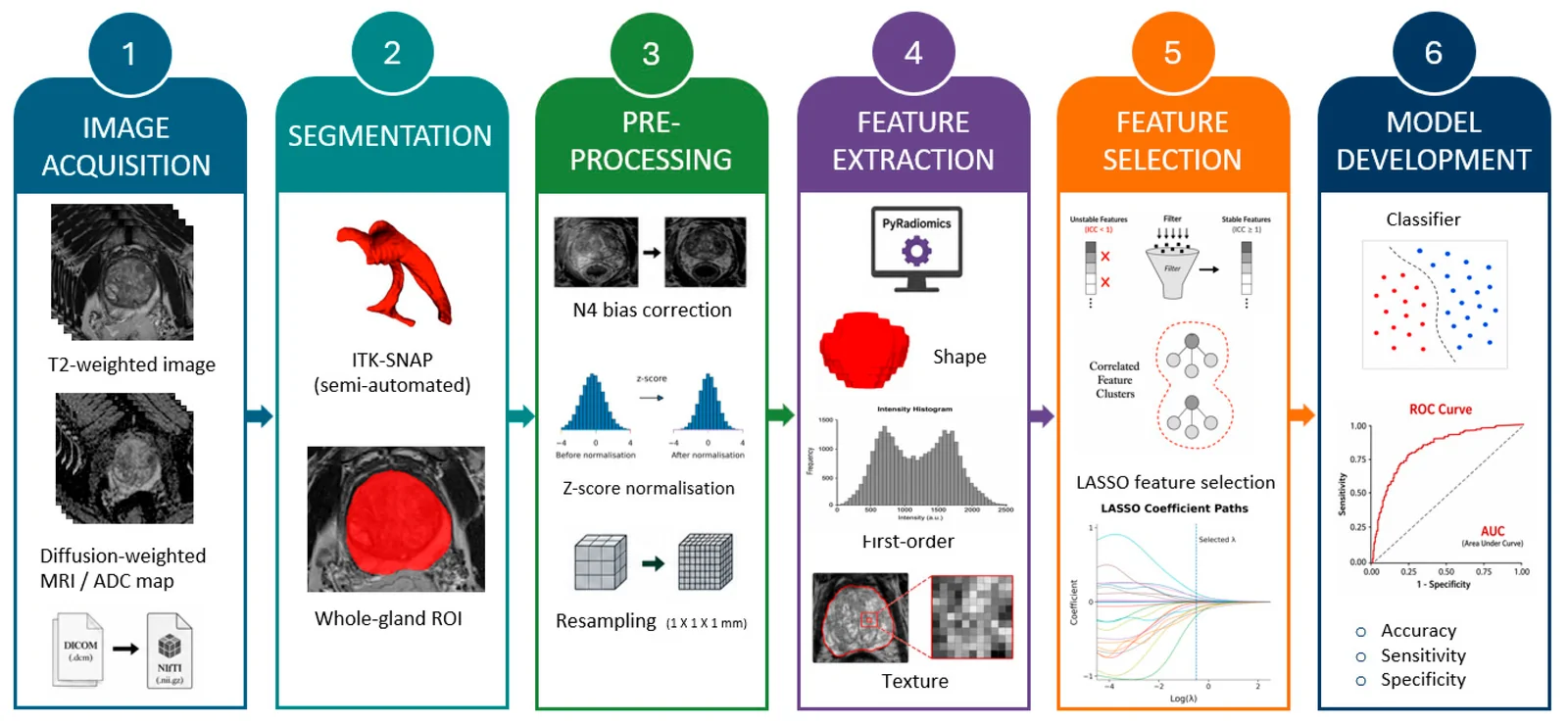
Overview of the radiomics workflow, including image acquisition, whole-gland segmentation, preprocessing, PyRadiomics feature extraction, feature selection, and model development.

The radiomics workflow is shown in Figure 2. Segmentation reproducibility was excellent in the 20-patient subset, with mean intra-reader and inter-reader Dice coefficients of 0.934 ± 0.028 and 0.914 ± 0.028, and corresponding HD95 values of 3.515 ± 1.466 mm and 4.039 ± 1.118 mm, respectively.

A total of 107 radiomic features were extracted per modality. After ICC-based stability filtering, 97 T2 and 80 ADC features were retained. Spearman correlation filtering reduced these to 28 T2 and 21 ADC non-redundant features. Across the five outer training folds, 27 T2 and 19 ADC radiomic features received a non-zero LASSO coefficient in at least one fold. Four T2 features and seven ADC features were selected in all five folds. To limit model complexity, the final retained radiomic predictors were restricted to one T2 feature, original_gldm_DependenceNonUniformityNormalized, and two ADC features, original_glcm_Imc1 and original_firstorder_RootMeanSquared (Table 2). These final fixed predictors were chosen from the features selected in all five outer folds, prioritising larger absolute LASSO coefficients, complementary feature classes, and model parsimony. They should be interpreted as quantitative imaging descriptors rather than direct biological markers without further validation.

### 3.3. Model Performance

**Figure 3:**
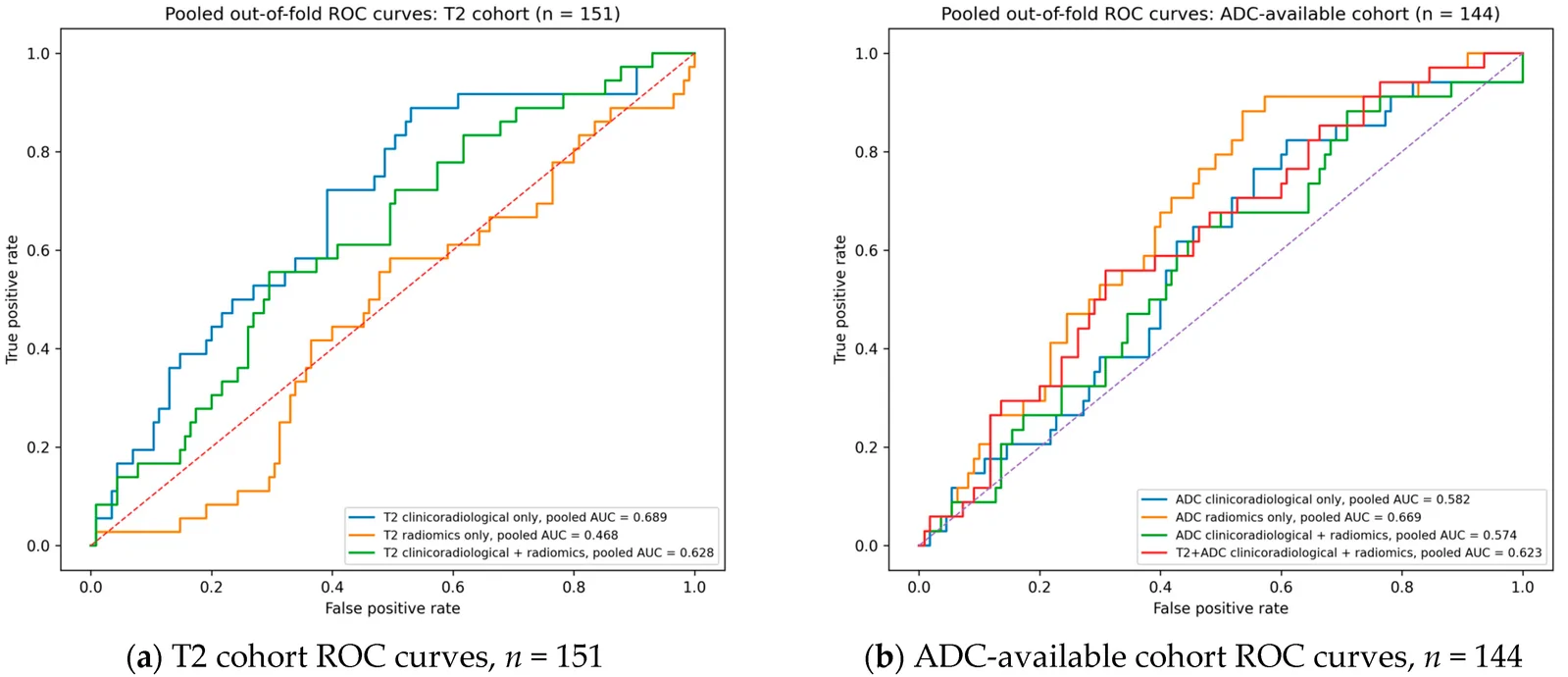
Pooled out-of-fold receiver operating characteristic (ROC) curves for the prediction models. (**a**) T2 cohort (*n* = 151; 36 subsequent csPCa detections), comparing the T2 clinicoradiological-only, T2 radiomics-only, and T2 clinicoradiological–radiomic models. (**b**) ADC-available cohort (*n* = 144; 34 subsequent csPCa detections), comparing the ADC clinicoradiological-only, ADC radiomics-only, ADC clinicoradiological–radiomic, and combined T2 + ADC clinicoradiological–radiomic models. Curves and AUC values shown in the figure were calculated from pooled out-of-fold predictions obtained from stratified five-fold cross-validation. These pooled AUC values differ from the mean fold-specific AUC ± SD values reported in Table 3. ADC, apparent diffusion coefficient; AUC, area under the receiver operating characteristic curve; ROC, receiver operating characteristic. The dashed diagonal lines represent the no-discrimination reference (AUC = 0.5).

Cross-validated model performance is summarised in Table 3, with ROC curves shown in Figure 3. Table 3 reports the mean fold-specific AUC across the five held-out folds, whereas Figure 3 shows pooled out-of-fold ROC curves and pooled AUC values calculated by combining predictions from all held-out folds. Therefore, the AUC values in Figure 3 differ slightly from the mean fold-specific AUC values reported in Table 3. In the T2 cohort, the T2 clinicoradiological–radiomic model achieved a mean AUC of 0.696 ± 0.060, an accuracy of 0.532 ± 0.165, a balanced accuracy of 0.602 ± 0.101, a sensitivity of 0.743 ± 0.310, a specificity of 0.461 ± 0.294, a precision of 0.309 ± 0.071, and an F1 score of 0.422 ± 0.104.

The T2 clinicoradiological-only model achieved a higher mean AUC of 0.713 ± 0.072 and higher balanced accuracy of 0.650 ± 0.100, compared with the T2 clinicoradiological–radiomic model. The T2 radiomics-only model showed poor discrimination, with a mean AUC of 0.456 ± 0.091 and balanced accuracy of 0.500 ± 0.000.

In the ADC-available cohort, the ADC clinicoradiological-only model achieved a mean AUC of 0.632 ± 0.193. The ADC radiomics-only model achieved a mean AUC of 0.684 ± 0.052, while the ADC clinicoradiological–radiomic and combined T2 + ADC clinicoradiological–radiomic models achieved mean AUCs of 0.635 ± 0.157 and 0.663 ± 0.106, respectively. Because T2-based models were evaluated in the T2 cohort and ADC-based models in the ADC-available cohort, comparisons between modalities are descriptive. Overall, radiomic features did not provide consistent incremental value over the clinicoradiological-only models. Comparisons of AUC and balanced accuracy across the main prediction models are shown in Supplementary Figure S1, while the corresponding sensitivity–specificity trade-offs are shown in Supplementary Figure S2. Paired comparison of pooled out-of-fold AUCs supported this interpretation. For T2, the clinicoradiological–radiomic model had a lower AUC than the clinicoradiological-only model (ΔAUC −0.062, 95% CI −0.111 to −0.017). Differences were smaller and uncertain for the ADC model (ΔAUC −0.009, 95% CI −0.082 to 0.064) and the combined T2 + ADC model (ΔAUC 0.041, 95% CI −0.041 to 0.127) (Supplementary Table S7).

### 3.4. Threshold and Sensitivity Analyses

For the primary T2 clinicoradiological–radiomic logistic-regression model, the default 0.50 threshold achieved high specificity but very low sensitivity. Compared with the fixed 0.50 threshold, the training-derived Youden threshold increased sensitivity from 0.086 ± 0.128 to 0.743 ± 0.310, but reduced specificity from 0.965 ± 0.057 to 0.461 ± 0.294 and reduced overall accuracy from 0.755 ± 0.016 to 0.532 ± 0.165 (Supplementary Table S1). This indicates that threshold tuning mainly shifted the sensitivity–specificity trade-off rather than producing uniformly improved classification performance.

In supplementary classifier comparisons using the same final T2 clinicoradiological–radiomic predictor set, logistic regression achieved an AUC of 0.696 ± 0.060, compared with 0.637 ± 0.157 for linear SVM and 0.652 ± 0.085 for the decision tree (Supplementary Table S2). In the class-weighted sensitivity analysis, the class-weighted logistic-regression model achieved a similar AUC to the unweighted model, with modestly higher balanced accuracy but lower sensitivity and higher specificity (Supplementary Table S3). Fold-by-fold performance of the T2 clinicoradiological–radiomic model using the training-derived Youden thresholds is reported in Supplementary Table S4. The pooled out-of-fold confusion matrix for the T2 clinicoradiological–radiomic model using the training-derived Youden thresholds is shown in Supplementary Figure S3. In the training-only feature-selection sensitivity analysis, mean AUCs were 0.662 ± 0.062 for the T2 model, 0.636 ± 0.136 for the ADC model, and 0.614 ± 0.139 for the combined T2 + ADC model (Supplementary Table S5). Feature selection varied across folds, particularly for T2 features (Supplementary Table S6), without altering the overall conclusion of limited incremental value from radiomics.

### 3.5. Calibration Assessment

Calibration of the primary T2 clinicoradiological–radiomic model was assessed using pooled out-of-fold predicted probabilities from stratified five-fold cross-validation. The model showed a pooled out-of-fold AUC of 0.628, a Brier score of 0.178, a calibration intercept of −0.451, and a calibration slope of 0.607. The calibration curve showed imperfect calibration, with approximate agreement in the lower predicted-risk range but deviations at higher predicted probabilities, including overprediction in the highest predicted-risk bin (Supplementary Figure S4).

## 4. Discussion

Patients with suspicious mpMRI findings but negative baseline biopsy represent a clinically challenging follow-up group, as a negative biopsy does not fully exclude csPCa and may reflect either false-positive MRI findings or false-negative biopsy sampling [21]. The main finding of this study was that baseline whole-gland MRI radiomics showed limited and inconsistent predictive value for subsequent csPCa detection. The T2 clinicoradiological–radiomic model achieved moderate discrimination but did not outperform the T2 clinicoradiological-only model. In particular, the clinicoradiological-only model achieved a higher mean AUC and better performance on most threshold-dependent metrics than the T2 clinicoradiological–radiomic model. The T2 radiomics-only model performed poorly, while the ADC-based and combined T2 + ADC models did not show consistent incremental value. These findings should be interpreted as exploratory and hypothesis-generating rather than evidence of clinical utility. The clinical setting distinguishes this study from most previous prostate MRI radiomics research. Previous studies have primarily evaluated baseline csPCa detection, PI-RADS 3 lesion classification, tumour grading, or outcomes in patients with confirmed prostate cancer [9,10,11,12,13,22,23], whereas the present study evaluated subsequent csPCa detection after suspicious mpMRI and negative baseline biopsy. This is a more complex outcome than baseline cancer detection, potentially reflecting missed baseline disease, biopsy sampling error, selective repeat investigation, and disease identified later during follow-up [21]. The modest performance may partly reflect the difficulty of this post-negative-biopsy setting, but it also indicates that the selected radiomic features were insufficient to improve prediction beyond clinicoradiological variables.

Baseline findings also highlighted the heterogeneity of this post-negative-biopsy population. Among patients with PI-RADS 5 lesions, 20 subsequently had csPCa detected, while 37 had no csPCa detected during available follow-up. Subsequent csPCa was also detected across lesion locations, including the peripheral zone (*n* = 12), transition zone (*n* = 15), and anterior fibromuscular stroma (*n* = 9). These findings suggest that neither PI-RADS category nor lesion location alone was sufficient to determine subsequent detection, although interpretation is limited by clinically driven, non-uniform follow-up and repeat biopsy.

Previous prostate MRI radiomics studies have reported variable performance, largely because of differences in cohort design, MRI sequences, segmentation strategy, clinical predictors, and reference standards. In the systematic review by Antolin et al. [9], handcrafted radiomics models reported internal-validation AUCs ranging from approximately 0.72 to 0.98. Several studies have evaluated lesion-based radiomics derived from T2-weighted, ADC/DWI, or combined MRI sequences and reported variable moderate-to-high discrimination, with performance differing substantially according to the model, cohort, and validation setting [24,25,26,27]. Some of them incorporated clinical predictors or PI-RADS into combined models, sometimes reporting higher discrimination [24,25,28]. Jing et al. [12] reported very high AUCs for a nomogram combining whole-prostate T2WI, lesion-based DWI radiomics, and PI-RADS. In the present study, PI-RADS was already incorporated within the clinicoradiological predictor set, whereas lesion-based DWI radiomics was not evaluated. However, that study used radical prostatectomy cohorts with a high prevalence of csPCa, whereas the present study included patients with negative baseline biopsy who were followed over time. These differences in clinical setting, outcome definition, event prevalence, and reference standard limit direct comparison.

The present findings are consistent with the cautious conclusions of Antolin et al. [9], who reported that prostate MRI radiomics shows promise but remains limited by methodological heterogeneity and inconsistent incremental value over established clinical or radiological assessment. In the present study, the T2 clinicoradiological-only model achieved a mean fold-specific AUC of 0.713 ± 0.072, while the T2 clinicoradiological–radiomic model achieved a mean fold-specific AUC of 0.696 ± 0.060. The clinicoradiological-only model also showed higher balanced accuracy, specificity, precision, and F1 score. This suggests that the selected T2 radiomic feature did not improve practical classification performance beyond established predictors such as PSA density, PI-RADS score, lesion location, and ASAP status. This distinction is important because a model may show moderate discrimination but still have limited clinical utility if threshold-dependent performance is poor.

The sequence-specific results were inconsistent and do not support a clear conclusion that one MRI sequence was superior. The T2 radiomics-only model performed poorly, with a mean AUC of 0.456 ± 0.091, whereas the ADC radiomics-only model showed moderate discrimination, with a mean AUC of 0.684 ± 0.052. However, the higher ADC radiomics-only AUC did not translate into clear incremental value when combined with clinicoradiological predictors, as the ADC clinicoradiological–radiomic model achieved a mean AUC of 0.635 ± 0.157, similar to the ADC clinicoradiological-only model. Because T2-based models were evaluated in the T2 cohort and ADC-based models in the smaller ADC-available cohort, these comparisons should be interpreted descriptively. ADC-derived radiomic features may also be influenced by DWI acquisition and processing factors, including b-value selection, scanner/vendor variability, and preprocessing or feature-extraction settings [29,30]. Although sequence-specific preprocessing and isotropic resampling were applied, the multicentre acquisition may have introduced scanner- or protocol-related batch effects that were not fully removed. As statistical harmonisation was not performed, residual acquisition-related variability cannot be excluded. Therefore, the sequence-specific findings remain exploratory and require external validation.

The whole-gland segmentation strategy is important when interpreting these findings. This approach was chosen because the clinical question was patient-level risk stratification after a negative biopsy, rather than classification of a single confirmed lesion. Previous prostate radiomics studies have most commonly used lesion-based segmentations, while whole-prostate and zonal approaches have been less frequently evaluated [9]. Jing et al. [12] directly compared lesion and whole-prostate segmentation strategies and reported that a model combining whole-prostate T2WI and lesion-based DWI achieved the best performance, supporting the importance of segmentation strategy in prostate radiomics. Because whole-gland radiomics is not restricted to the suspicious lesion, averaging imaging information across the entire prostate may dilute lesion-specific signals by combining the suspicious lesion with benign and background prostate tissue. At the same time, whole-gland features may reflect broader gland-level heterogeneity, but may also capture benign prostatic changes, background tissue composition, scanner-related variation, and preprocessing effects [29,30,31]. The selected features represented different aspects of image texture and intensity. The T2-derived GLDM DependenceNonUniformityNormalized feature describes the homogeneity of dependence patterns within the segmented region, while the ADC-derived GLCM Imc1 feature reflects statistical dependence between grey-level probability distributions, and the first-order RootMeanSquared feature reflects the overall magnitude of voxel intensities [32]. These features should be interpreted as quantitative imaging descriptors rather than direct biological markers of tumour aggressiveness without further biological validation [33]. These factors may help explain why some radiomic features showed predictive signals when considered alone, particularly in the ADC analysis, but did not provide consistent incremental information once established clinicoradiological predictors were included. In this setting, much of the clinically relevant risk information may already have been represented by PSA density, PI-RADS score, lesion location, and ASAP status, while whole-gland radiomic features may have added predominantly non-specific imaging variation. Future studies should compare whole-gland, lesion-based, zonal, and peri-lesional radiomics in larger prospective multicentre cohorts of patients with suspicious mpMRI and negative biopsy. Standardised imaging and follow-up protocols, fully nested model development, and independent external validation are needed to determine the most informative segmentation strategy and assess whether radiomics provides reproducible incremental value beyond clinicoradiological predictors.

The threshold analysis supports cautious clinical interpretation. Compared with the fixed 0.50 threshold, the training-derived Youden threshold increased sensitivity from 0.086 ± 0.128 to 0.743 ± 0.310 and increased balanced accuracy from 0.525 ± 0.036 to 0.602 ± 0.101. However, this came at the cost of substantially lower specificity, which decreased from 0.965 ± 0.057 to 0.461 ± 0.294, and lower overall accuracy, which decreased from 0.755 ± 0.016 to 0.532 ± 0.165. This indicates that threshold optimisation mainly shifted the sensitivity–specificity trade-off rather than producing uniformly improved classification performance. Although higher sensitivity may be useful in follow-up settings to avoid missing csPCa, the modest precision of the Youden-threshold model suggests that many higher-risk classifications would be false positives. Therefore, the model is not ready to guide repeat biopsy decisions alone.

Calibration findings also support cautious interpretation. The calibration slope was below 1, suggesting that predicted risks were not perfectly calibrated, and the calibration curve showed deviation from the ideal line, particularly in the higher predicted-risk range. The model may therefore provide limited risk-ranking information, but its predicted probabilities should not be used for individual clinical decision-making without recalibration and external validation. The fold-specific results showed appreciable variability in discrimination, sensitivity, specificity, and selected thresholds. With only 36 csPCa events in the T2 cohort, each validation fold contained approximately seven events, meaning that the classification of a small number of patients could materially affect the estimated performance. The selected features and model performance may therefore be sensitive to data partitioning, limiting confidence that the same predictors and performance would be reproduced in a new cohort.

This post-negative-biopsy follow-up setting represents the principal novelty of the study and differs from the baseline diagnostic and lesion-characterisation settings examined in most previous prostate radiomics research. The radiomics workflow included segmentation reproducibility assessment, ICC-based stability filtering, correlation filtering, outer-fold LASSO-based feature prioritisation, and cross-validated model evaluation. Standardisation, LASSO fitting, and regularisation-parameter tuning were restricted to the outer training folds during feature selection, while threshold optimisation was performed using training data during final model evaluation. The primary analysis used unweighted logistic regression, with class-weighted logistic regression evaluated as a sensitivity analysis. Supplementary classifier comparisons using linear SVM and a shallow decision tree showed broadly similar findings. These steps improve methodological transparency and reduce the risk of data leakage, but they do not overcome the limited number of csPCa events, the exploratory feature-selection workflow, or the lack of external validation.

Several limitations should be acknowledged. First, the sample size was modest, with only 36 csPCa events, limiting model complexity and increasing uncertainty in performance estimates. Although LASSO tuning and initial feature selection were performed within nested cross-validation, selection frequencies across all outer folds were subsequently used to define fixed parsimonious feature sets for final model development. The final performance evaluation was therefore not fully independent of feature definition, and some optimism in the reported estimates cannot be excluded. However, the training-only feature-selection sensitivity analysis produced similar or lower discrimination and did not alter the overall conclusion. Second, follow-up was clinically driven rather than protocolised, which may have introduced detection bias; therefore, the outcome reflects clinically detected csPCa rather than true csPCa prevalence. Third, early csPCa detection suggests that outcomes may include missed baseline disease, biopsy sampling error, and disease detected later during follow-up. Fourth, whole-gland radiomics may capture benign prostatic changes, background tissue variation, scanner effects, or preprocessing influences rather than tumour-specific features. Fifth, a sensitivity analysis excluding early csPCa detections was not performed because of the limited total number of outcome events and the concentration of detections early in follow-up, which would have substantially reduced the number of events available for modelling and further destabilised performance estimates. Finally, calibration was assessed internally and may be unreliable given the small number of events. Several of these limitations, including the limited event count, clinically driven follow-up, acquisition heterogeneity, and absence of an independent validation cohort, are inherent to the retrospective design and could not be fully resolved within the present study. External validation in a larger, independently assembled cohort with standardised imaging and follow-up is therefore required before clinical application.

## 5. Conclusions

Baseline whole-gland MRI radiomics showed limited and inconsistent predictive value for subsequent csPCa detection in patients with suspicious mpMRI findings and negative baseline biopsy. The addition of selected radiomic features did not demonstrate consistent incremental improvement over clinicoradiological predictors alone. These findings are exploratory and hypothesis-generating, and external validation in larger, independent cohorts is required before any clinical application.

## Figures and Tables

**Table 1:** Baseline and follow-up characteristics of the study cohort stratified by subsequent csPCa detection status.

Baseline Variables	Overall Cohort	Subsequent csPCa Detected	No Subsequent csPCa Detected	*p*-Value
Number of patients (%)	151 (100%)	36 (23.8%)	115 (76.2%)	-
Age (years), median (IQR)	65.00 (60.00–69.00)	68.00 (61.75–70.00)	65.00 (59.00–69.00)	0.097
PSA level (ng/mL), median (IQR)	7.55 (5.90–9.90)	8.35 (6.15–10.62)	7.40 (5.83–9.57)	0.296
Prostate volume (mL), median (IQR)	57.00 (38.50–75.00)	43.00 (35.00–62.75)	62.00 (45.00–79.50)	0.016
PSA density (ng/mL/cm^3^), median (IQR)	0.13 (0.08–0.19)	0.17 (0.10–0.25)	0.12 (0.08–0.18)	0.011
PI-RADS 3	29 (19.2%)	6 (16.7%)	23 (20.0%)	0.035
PI-RADS 4	65 (43.0%)	10 (27.8%)	55 (47.8%)	-
PI-RADS 5	57 (37.7%)	20 (55.6%)	37 (32.2%)	-
Index lesion location	-	-	-	-
Peripheral zone (PZ)	89 (58.9%)	12 (33.3%)	77 (67.0%)	<0.001
Transition zone (TZ)	46 (30.5%)	15 (41.7%)	31 (27.0%)	-
Anterior fibromuscular stroma (AFS)	16 (10.6%)	9 (25.0%)	7 (6.1%)	-
ASAP present, yes	14 (9.3%)	7 (19.4%)	7 (6.1%)	0.042
Follow-up variables	-	-	-	-
Time to csPCa detection or last follow-up, months, median (IQR)	64.00 (15.00–84.50)	3.00 (2.00–6.00)	72.00 (57.00–89.00)	-
Patients undergoing baseline plus ≥1 repeat biopsy procedure, *n* (%)	82 (54.3%)	36 (100.0%)	46 (40.0%)	-
Patients undergoing baseline plus ≥1 repeat mpMRI scan, *n* (%)	56 (37.1%)	15 (41.7%)	41 (35.7%)	-

Data are presented as median (IQR) or *n* (%). *p*-values are presented for baseline variables only and compare patients with and without subsequent csPCa detection using the Mann–Whitney U test, chi-square test, or Fisher’s exact test, as appropriate. PI-RADS score and lesion location were analysed as grouped categorical variables. Follow-up variables are presented descriptively because they occurred after baseline and were influenced by the subsequent clinical and outcome-ascertainment pathway. AFS, anterior fibromuscular stroma; ASAP, atypical small acinar proliferation; csPCa, clinically significant prostate cancer; mpMRI, multiparametric MRI; PI-RADS, Prostate Imaging Reporting and Data System; PSA, prostate-specific antigen; PZ, peripheral zone; TZ, transition zone.

**Table 2:** Feature-selection summary.

Modality	Initial Extracted Radiomic Features	ICC-Stable	Non-Redundant	Candidate Features with Non-Zero LASSO Coefficients	Features Selected in all Five Folds	Final Retained	Final Radiomic Features Retained for Modelling	Folds Selected
T2	107	97	28	27	4	1	original_gldm_DependenceNonUniformityNormalized	5/5
ADC	107	80	21	19	7	2	original_glcm_Imc1; original_firstorder_RootMeanSquared	5/5; 5/5

Candidate features were defined as radiomic features receiving a non-zero LASSO coefficient in at least one outer training fold. Final retained features were chosen from features selected in all five outer folds, prioritising larger absolute LASSO coefficients, complementary feature class, and model parsimony. ICC, intraclass correlation coefficient; LASSO, least absolute shrinkage and selection operator; ADC, apparent diffusion coefficient.

**Table 3:** Performance of clinicoradiological-only, radiomics-only, and clinicoradiological–radiomic models in the T2 and ADC-available cohorts.

Model	Cohort Size, *n*	AUC, Mean ± SD	Pooled OOF AUC (95% CI)	Accuracy, Mean ± SD	Balanced Accuracy, Mean ± SD	Precision, Mean ± SD	Sensitivity, Mean ± SD	Specificity, Mean ± SD	Youden Index, Mean ± SD	F1 Score, Mean ± SD
T2 clinicoradiological–radiomic	151	0.696 ± 0.060	0.628 (0.525–0.726)	0.532 ± 0.165	0.602 ± 0.101	0.309 ± 0.071	0.743 ± 0.310	0.461 ± 0.294	0.204 ± 0.202	0.422 ± 0.104
T2 radiomics-only	151	0.456 ± 0.091	0.468 (0.364–0.574)	0.548 ± 0.288	0.500 ± 0.000	0.093 ± 0.128	0.400 ± 0.548	0.600 ± 0.548	0.000 ± 0.000	0.151 ± 0.207
T2 clinicoradiological-only	151	0.713 ± 0.072	0.689 (0.589–0.783)	0.642 ± 0.106	0.650 ± 0.100	0.372 ± 0.125	0.664 ± 0.242	0.635 ± 0.167	0.299 ± 0.200	0.465 ± 0.137
ADC clinicoradiological-only	144	0.632 ± 0.193	0.582 (0.476–0.686)	0.541 ± 0.090	0.603 ± 0.145	0.298 ± 0.105	0.724 ± 0.268	0.482 ± 0.069	0.206 ± 0.289	0.422 ± 0.150
ADC radiomics-only	144	0.684 ± 0.052	0.669 (0.571–0.762)	0.612 ± 0.067	0.580 ± 0.043	0.310 ± 0.022	0.524 ± 0.205	0.636 ± 0.140	0.160 ± 0.086	0.380 ± 0.058
ADC clinicoradiological–radiomic	144	0.635 ± 0.157	0.574 (0.466–0.680)	0.597 ± 0.118	0.593 ± 0.066	0.439 ± 0.318	0.586 ± 0.278	0.600 ± 0.228	0.186 ± 0.131	0.384 ± 0.105
T2 + ADC clinicoradiological–radiomic	144	0.663 ± 0.106	0.623 (0.518–0.725)	0.583 ± 0.100	0.594 ± 0.077	0.321 ± 0.090	0.614 ± 0.096	0.573 ± 0.131	0.187 ± 0.153	0.416 ± 0.084

Values are reported as mean ± SD across the five held-out folds of stratified five-fold cross-validation. Pooled out-of-fold (OOF) AUCs were calculated by combining predictions from all held-out outer folds; 95% confidence intervals were estimated using 20,000 stratified patient-level bootstrap resamples. T2 models were evaluated in the T2 cohort (*n* = 151; 36 subsequent csPCa detections), whereas ADC and combined T2 + ADC models were evaluated in the ADC-available cohort (*n* = 144; 34 subsequent csPCa detections). Threshold-dependent metrics were calculated using fold-specific thresholds optimised within the corresponding training folds. ADC, apparent diffusion coefficient; AUC, area under the receiver operating characteristic curve; csPCa, clinically significant prostate cancer; OOF, out of fold; SD, standard deviation.

## Data Availability

The datasets generated and analysed during the current study are not publicly available due to privacy and ethical restrictions, as they contain sensitive patient clinical and MRI data. Requests to access the data should be directed to the corresponding author and will be considered subject to institutional approval and appropriate data-sharing agreements.

## Supplementary Materials

The following supporting information can be downloaded at: https://www.mdpi.com/article/10.3390/cancers18172823/s1, Supplementary Figure S1: Comparison of AUC and balanced accuracy across the main prediction models; Supplementary Figure S2: Sensitivity and specificity trade-off across the main prediction models; Supplementary Figure S3: Pooled out-of-fold confusion matrix for the T2 clinicoradiological–radiomic model using training-derived Youden thresholds; Supplementary Figure S4: Calibration plot for the primary T2 clinicoradiological–radiomic model; Supplementary Table S1: Fixed 0.50 versus training-derived Youden threshold for the T2 clinicoradiological–radiomic logistic-regression model; Supplementary Table S2: Classifier comparison for the T2 clinicoradiological–radiomic model; Supplementary Table S3: Unweighted versus class-weighted T2 clinicoradiological–radiomic logistic-regression model; Supplementary Table S4: Fold-by-fold performance of the T2 clinicoradiological–radiomic model using training-derived Youden thresholds; Supplementary Table S5: Training-only radiomic feature-selection sensitivity analysis; Supplementary Table S6: Radiomic feature selection across outer training folds in the training-only sensitivity analysis; Supplementary Table S7: Paired comparisons of pooled out-of-fold AUCs for incremental radiomic value; Supplementary File S1: Sequence-specific PyRadiomics YAML configuration files for T2-weighted and ADC radiomic feature extraction.

## Abbreviations

The following abbreviations are used in this manuscript:

ADC	Apparent diffusion coefficient
ASAP	Atypical small acinar proliferation
AUC	Area under the receiver operating characteristic curve
CLEAR	CheckList for EvaluAtion of Radiomics research
GLCM	Grey-level co-occurrence matrix
GLRLM	Grey-level run-length matrix
GLSZM	Grey-level size-zone matrix
GLDM	Grey-level dependence matrix
ICC	Intraclass correlation coefficient
LASSO	Least absolute shrinkage and selection operator
NGTDM	Neighbouring grey-tone difference matrix
PI-RADS	Prostate Imaging Reporting and Data System
PSA	Prostate-specific antigen
ROC	Receiver operating characteristic
TRIPOD + AI	Transparent Reporting of a multivariable prediction model for Individual Prognosis Or Diagnosis + Artificial Intelligence
