# Supplementary material for: Baseline Whole-Gland MRI Radiomics for Risk Stratification After Suspicious mpMRI and Negative Biopsy: A MULTIPROS Sub-Analysis
Source: Cancers (Basel). 2026 Sep 1;18(17):2823. doi: 10.3390/cancers18172823 (PMC13565514; doi:10.3390/cancers18172823)
Supplement: Supplementary file 1 [file cancers-18-02823-s001.zip › cancers-4507515-supplementary.pdf]

Supplementary Materials

Baseline whole-gland MRI radiomics for risk stratification after suspicious mpMRI and negative biopsy: a MULTIPROS sub-analysis

1. Supplementary Tables:

Supplementary Table S1. Fixed 0.50 versus training-derived Youden threshold for the T2 clinicoradiological-radiomic logistic-regression model.

Threshold	AUC, mean ± SD	Accuracy, mean ± SD	Balanced accuracy, mean ± SD	PPV, mean ± SD	Sensitivity, mean ± SD	Specificity, mean ± SD	Youden index, mean ± SD	F1 score, mean ± SD	NPV, mean ± SD
**Fixed 0.50**	0.696 ± 0.060	0.755 ± 0.016	0.525 ± 0.036	0.450 ± 0.071*	0.086 ± 0.128	0.965 ± 0.057	0.051 ± 0.072	0.111 ± 0.157	0.772 ± 0.022
**Training-derived Youden**	0.696 ± 0.060	0.532 ± 0.165	0.602 ± 0.101	0.309 ± 0.071	0.743 ± 0.310	0.461 ± 0.294	0.204 ± 0.202	0.422 ± 0.104	0.880 ± 0.100†

**PPV and NPV were calculated for folds in which the corresponding predictive value was defined. PPV was undefined in three folds at the fixed 0.50 threshold because no patients were classified as positive; the reported mean ± SD was calculated across the two evaluable folds. †NPV was undefined in one fold at the training-derived Youden threshold because no patients were classified as negative; the reported mean ± SD was calculated across the four evaluable folds. PPV, positive predictive value; NPV, negative predictive value.*

Abbreviations: AUC, area under the receiver operating characteristic curve; SD, standard deviation.

Supplementary Table S2. Classifier comparison for the T2 clinicoradiological-radiomic model.

Classifier	AUC, mean ± SD	Accuracy, mean ± SD	Balanced accuracy, mean ± SD	Precision, mean ± SD	Sensitivity, mean ± SD	Specificity, mean ± SD	Youden index, mean ± SD	F1 score, mean ± SD
**Logistic regression**	0.696 ± 0.060	0.532 ± 0.165	0.602 ± 0.101	0.309 ± 0.071	0.743 ± 0.310	0.461 ± 0.294	0.204 ± 0.202	0.422 ± 0.104
**Linear SVM**	0.637 ± 0.157	0.495 ± 0.261	0.551 ± 0.048	0.362 ± 0.183	0.650 ± 0.368	0.452 ± 0.458	0.102 ± 0.096	0.371 ± 0.025

Classifier	AUC, mean ± SD	Accuracy, mean ± SD	Balanced accuracy, mean ± SD	Precision, mean ± SD	Sensitivity, mean ± SD	Specificity, mean ± SD	Youden index, mean ± SD	F1 score, mean ± SD
**Decision tree**	0.652 ± 0.085	0.643 ± 0.102	0.596 ± 0.088	0.356 ± 0.098	0.514 ± 0.344	0.678 ± 0.221	0.193 ± 0.176	0.374 ± 0.136

Abbreviations: AUC, area under the receiver operating characteristic curve; SD, standard deviation; SVM, support vector machine.

Supplementary Table S3. Unweighted versus class-weighted T2 clinicoradiological-radiomic logistic-regression model.

Imbalance method	AUC, mean ± SD	Accuracy, mean ± SD	Balanced accuracy, mean ± SD	Precision, mean ± SD	Sensitivity, mean ± SD	Specificity, mean ± SD	Youden index, mean ± SD	F1 score, mean ± SD
**Unweighted**	0.696 ± 0.060	0.532 ± 0.165	0.602 ± 0.101	0.309 ± 0.071	0.743 ± 0.310	0.461 ± 0.294	0.204 ± 0.202	0.422 ± 0.104
**Class-weighted**	0.707 ± 0.060	0.589 ± 0.092	0.615 ± 0.075	0.325 ± 0.077	0.664 ± 0.242	0.565 ± 0.168	0.230 ± 0.149	0.426 ± 0.104

Abbreviations: AUC, area under the receiver operating characteristic curve; SD, standard deviation.

Supplementary Table S4. Fold-by-fold performance of the T2 clinicoradiological-radiomic model using training-derived Youden thresholds.

Fold	Best threshold	AUC	Accuracy	Balanced accuracy	Sensitivity	Specificity	Precision	F1 score	TN	FP	FN	TP
**1**	0.228	0.766	0.258	0.500	1.000	0.000	0.258	0.410	0	23	0	8
**2**	0.208	0.615	0.600	0.491	0.286	0.696	0.222	0.250	16	7	5	2
**3**	0.199	0.658	0.567	0.717	1.000	0.435	0.350	0.519	10	13	0	7

Fold	Best threshold	AUC	Accuracy	Balanced accuracy	Sensitivity	Specificity	Precision	F1 score	TN	FP	FN	TP
**4**	0.241	0.727	0.700	0.655	0.571	0.739	0.400	0.471	17	6	3	4
**5**	0.172	0.714	0.533	0.646	0.857	0.435	0.316	0.462	10	13	1	6
**Mean ± SD**	0.210 ± 0.027	0.696 ± 0.060	0.532 ± 0.165	0.602 ± 0.101	0.743 ± 0.310	0.461 ± 0.294	0.309 ± 0.071	0.422 ± 0.104	10.600 ± 6.768	12.400 ± 6.768	1.800 ± 2.168	5.400 ± 2.408

Abbreviations: AUC, area under the receiver operating characteristic curve; FN, false negative; FP, false positive; SD, standard deviation; TN, true negative; TP, true positive.

Supplementary Table S5. Training-only radiomic feature-selection sensitivity analysis.

Model	Cohort, n (events)	Clinicoradiological-only AUC, mean ± SD	Primary fixed-feature model AUC, mean ± SD	Training-only feature-selection sensitivity AUC, mean ± SD
**T2 clinicoradiological-radiomic**	151 (36)	0.713 ± 0.072	0.696 ± 0.060	**0.662 ± 0.062**
**ADC clinicoradiological-radiomic**	144 (34)	0.632 ± 0.193	0.635 ± 0.157	**0.636 ± 0.136**
**T2+ADC clinicoradiological-radiomic**	144 (34)	0.632 ± 0.193*	0.663 ± 0.106	**0.614 ± 0.139**

*Values are mean ± SD across the five held-out outer folds. In the training-only feature-selection sensitivity analysis, radiomic feature selection was repeated using only the corresponding outer training data before evaluation in the held-out outer fold. The same parsimonious dimensionality as the primary models was retained: one T2 feature and two ADC features. *The ADC clinicoradiological-only model provides the clinicoradiological comparator for the combined T2+ADC model because both were evaluated in the same ADC-available cohort (n = 144; 34 subsequent csPCa detections). AUC, area under the receiver operating characteristic curve; ADC, apparent diffusion coefficient; SD, standard deviation.*

Supplementary Table S6. Radiomic feature selection across outer training folds in the training-only sensitivity analysis.

Model / modality	Radiomic feature	Folds selected
**T2 model**	original_firstorder_Kurtosis	3/5
	original_firstorder_Median	1/5
	original_glrlm_RunEntropy	1/5
**ADC model**	original_glcm_Imc1	5/5
	original_gldm_SmallDependenceHighGrayLevelEmphasis	2/5
	original_glszm_ZoneEntropy	2/5
	original_firstorder_RootMeanSquared	1/5
**Combined T2+ADC model – T2 component**	original_firstorder_Kurtosis	3/5
	original_gldm_DependenceNonUniformityNormalized	1/5
	original_shape_Maximum2DDiameterSlice	1/5
**Combined T2+ADC model – ADC component**	original_glcm_Imc1	5/5
	original_gldm_SmallDependenceHighGrayLevelEmphasis	2/5
	original_glszm_ZoneEntropy	2/5
	original_firstorder_RootMeanSquared	1/5

Feature selection was repeated independently within each outer training fold. One T2 feature and two ADC features with the largest absolute non-zero standardised LASSO coefficients were retained within each fold. For the combined T2+ADC model, T2 and ADC features were selected separately within the same outer training fold. Selection frequencies therefore describe the stability of training-only feature selection across the five outer folds.

Supplementary Table S7. Paired comparisons of pooled out-of-fold AUCs for incremental radiomic value.

Comparison	AUC model A	AUC model B	ΔAUC (B−A)	95% CI for ΔAUC
**T2 clinicoradiological-only vs T2 clinicoradiological-radiomic**	0.689	0.628	−0.062	−0.111 to −0.017
**ADC clinicoradiological-only vs ADC clinicoradiological-radiomic**	0.582	0.574	−0.009	−0.082 to 0.064
**ADC clinicoradiological-only vs T2+ADC clinicoradiological-radiomic**	0.582	0.623	0.041	−0.041 to 0.127

Differences are calculated as model B minus model A. Confidence intervals were estimated using 20,000 paired stratified patient-level bootstrap resamples of pooled out-of-fold predictions. Only models evaluated within the same cohort were formally compared.

2. Supplementary Figures:

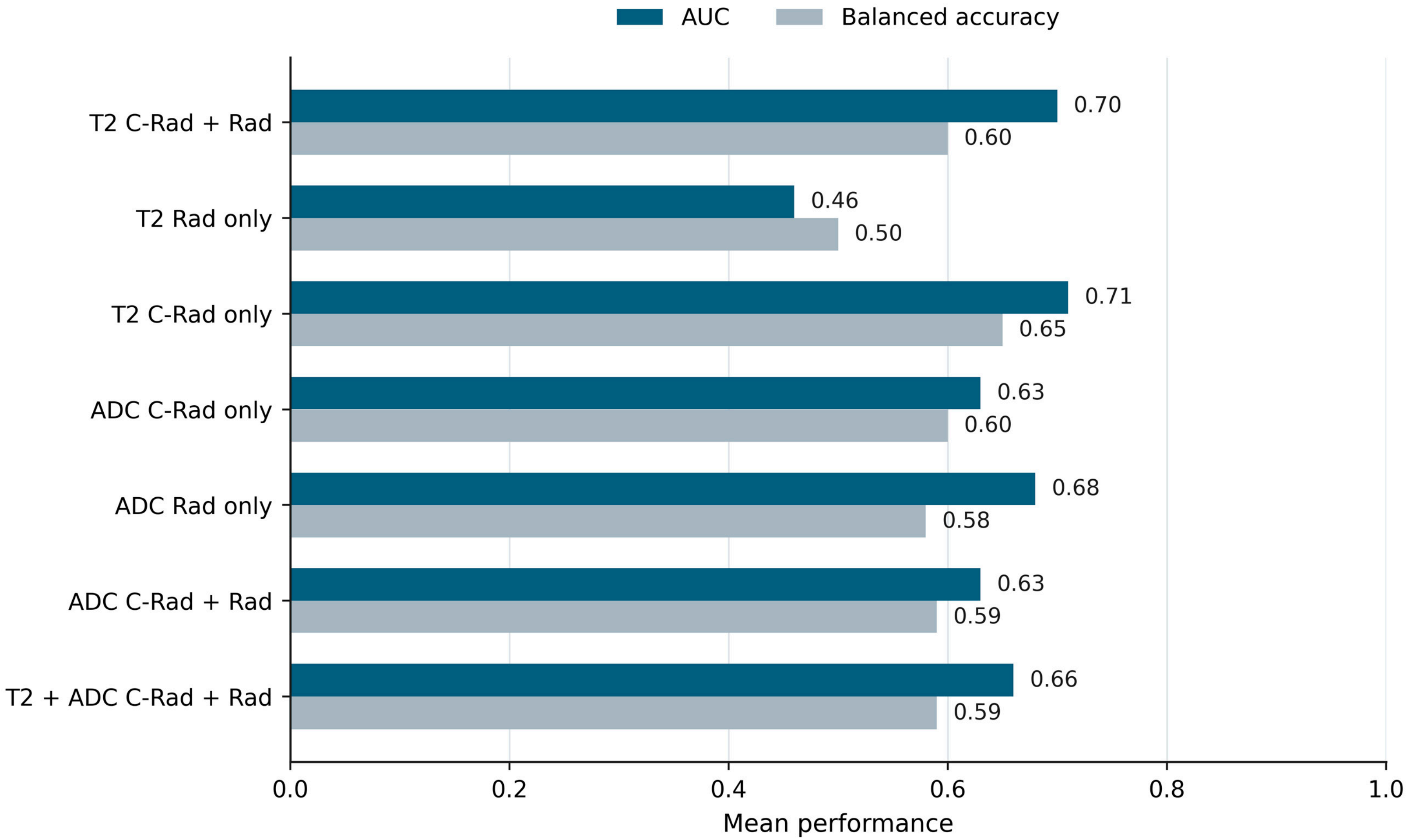

Supplementary Figure S1. Comparison of AUC and balanced accuracy across the main prediction models. Bars show mean values across the five held-out folds of stratified five-fold cross-validation. Corresponding mean ± SD values are provided in Table 3.

Abbreviations: ADC, apparent diffusion coefficient; AUC, area under the receiver operating characteristic curve; C-Rad, clinicoradiological; Rad, radiomics; SD, standard deviation; T2, T2-weighted imaging.

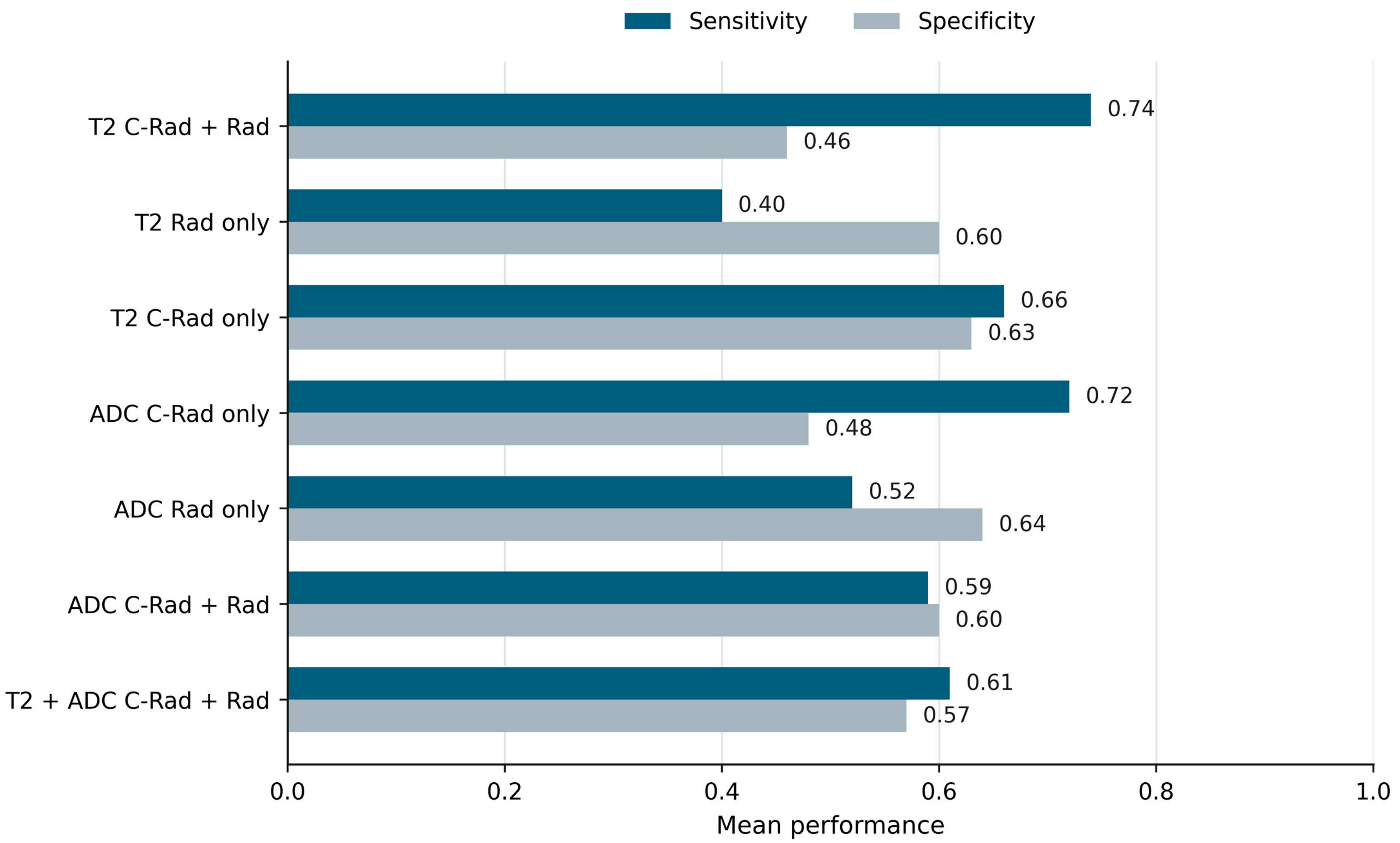

Supplementary Figure S2. Sensitivity and specificity trade-off across the main prediction models. Bars show mean values across the five held-out folds of stratified five-fold cross-validation. Corresponding mean ± SD values are provided in Table 3.

Abbreviations: ADC, apparent diffusion coefficient; C-Rad, clinicoradiological; Rad, radiomics; SD, standard deviation; T2, T2-weighted imaging.

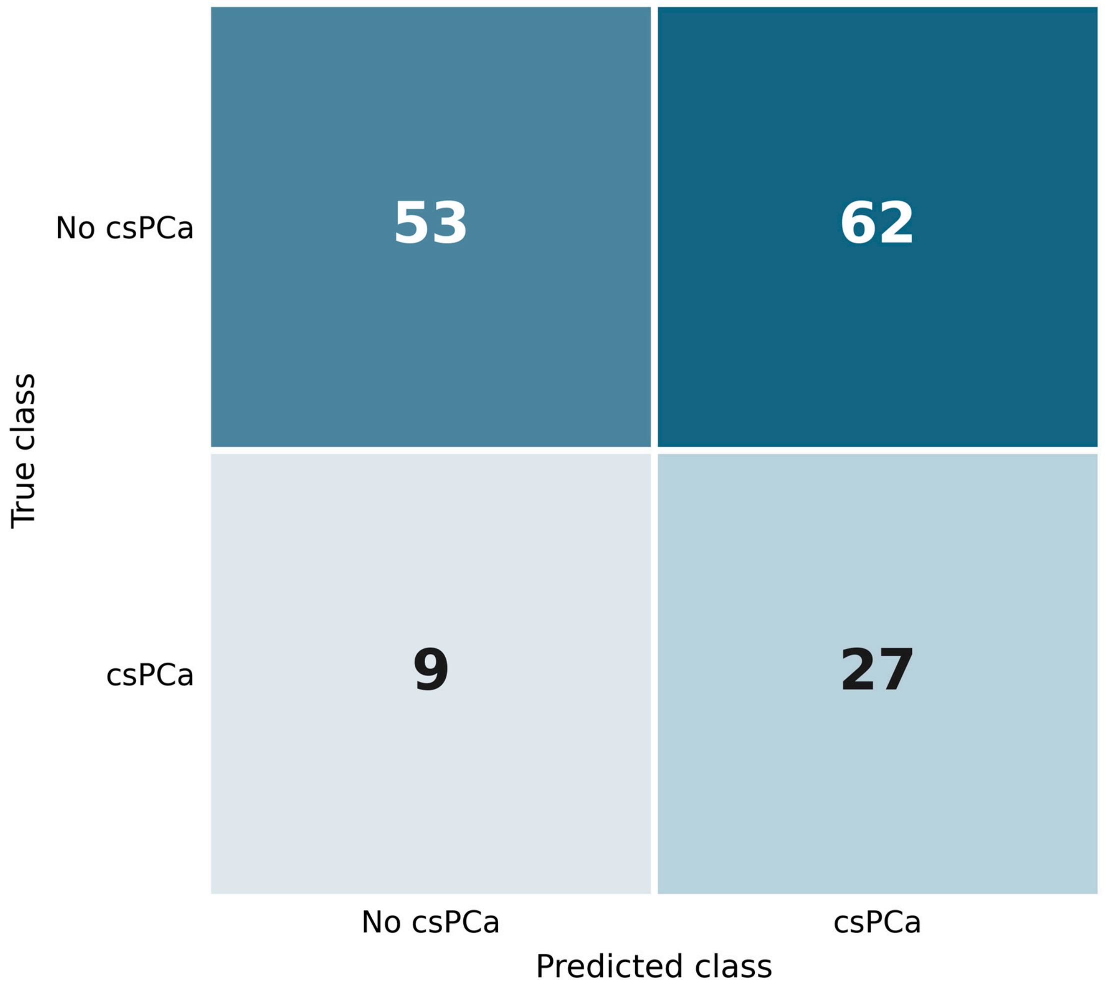

Supplementary Figure S3. Pooled out-of-fold confusion matrix for the T2 clinicoradiological-radiomic model using training-derived Youden thresholds. Predictions were generated from the held-out outer test folds of stratified five-fold cross-validation. The fold-specific training-derived Youden threshold was applied to each corresponding held-out fold. The mean threshold across folds was 0.210 ± 0.027. The corresponding mean fold-specific performance estimates were sensitivity of 0.743 ± 0.310, specificity of 0.461 ± 0.294, precision/positive predictive value of 0.309 ± 0.071, and F1 score of 0.422 ± 0.104. The outcome prevalence in the T2 cohort was 36/151 (23.8%).

Abbreviations: csPCa, clinically significant prostate cancer; T2, T2-weighted imaging.

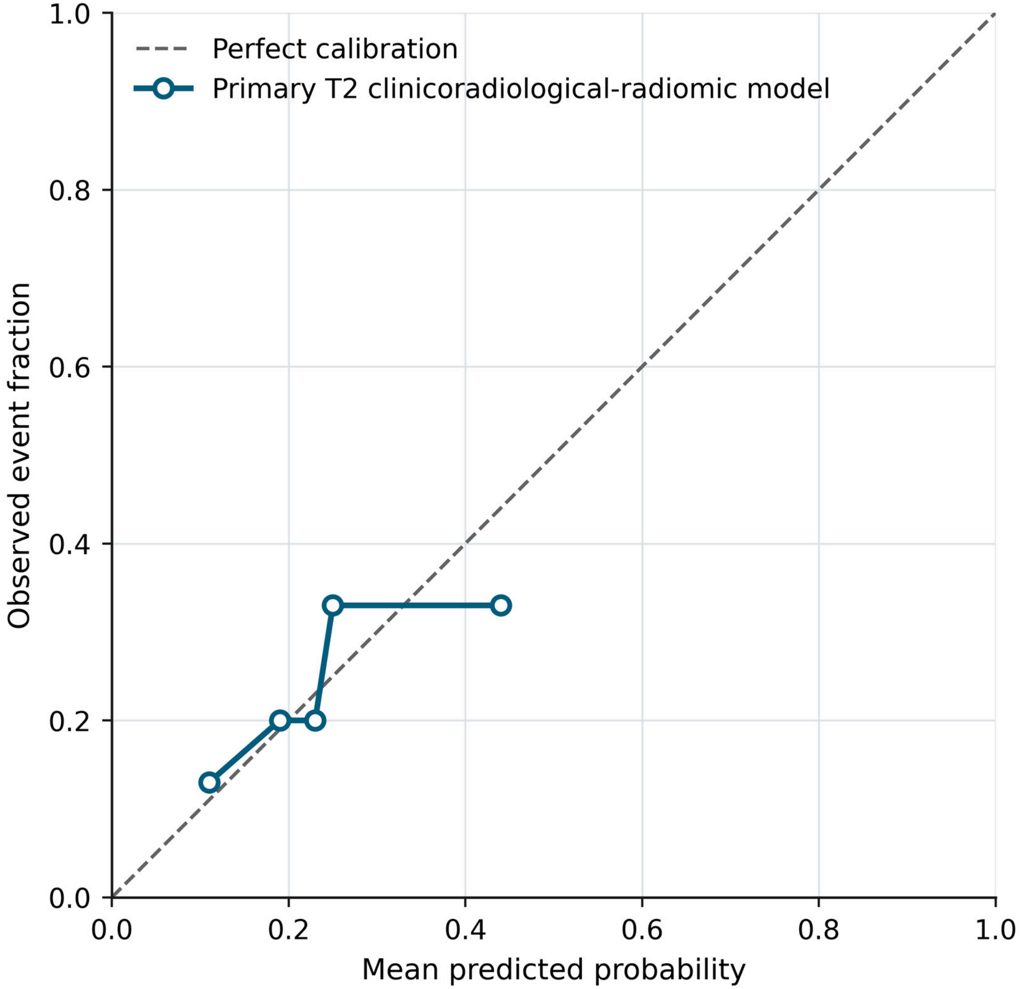

Supplementary Figure S4. Calibration plot for the primary T2 clinicoradiological-radiomic model. Calibration was assessed using pooled out-of-fold predicted probabilities from stratified five-fold cross-validation. Points represent five quantile-based calibration bins, and the dashed diagonal line represents perfect calibration. The model showed imperfect calibration, with approximate agreement in the lower predicted-risk range but deviations at higher predicted probabilities, including overprediction in the highest predicted-risk bin.

Abbreviation: T2, T2-weighted imaging.
